# Suicidal Behavior and Depression in Smoking Cessation Treatments

**DOI:** 10.1371/journal.pone.0027016

**Published:** 2011-11-02

**Authors:** Thomas J. Moore, Curt D. Furberg, Joseph Glenmullen, John T. Maltsberger, Sonal Singh

**Affiliations:** 1 Institute for Safe Medication Practices, Alexandria, Virginia, United States of America; 2 Division of Public Health Sciences, Wake Forest University School of Medicine, Winston-Salem, North Carolina, United States of America; 3 Department of Psychiatry-Cambridge Hospital, Harvard Medical School, Cambridge, Massachusetts, United States of America; 4 Department of Psychiatry-McLean Hospital, Harvard Medical School, Belmont, Massachusetts, United States of America; 5 Department of Medicine, Johns Hopkins University School of Medicine, Baltimore, Maryland, United States of America; University of British Columbia, Canada

## Abstract

**Background:**

Two treatments for smoking cessation—varenicline and bupropion—carry Boxed Warnings from the U.S. Food and Drug Administration (FDA) about suicidal/self-injurious behavior and depression. However, some epidemiological studies report an increased risk in smoking or smoking cessation independent of treatment, and differences between drugs are unknown.

**Methodology:**

From the FDA's Adverse Event Reporting System (AERS) database from 1998 through September 2010 we selected domestic, serious case reports for varenicline (n = 9,575), bupropion for smoking cessation (n = 1,751), and nicotine replacement products (n = 1,917). A composite endpoint of suicidal/self-injurious behavior or depression was defined as a case with one or more Preferred Terms in Standardized MedDRA Query (SMQ) for those adverse effects. The main outcome measure was the ratio of reported suicide/self-injury or depression cases for each drug compared to all other serious events for that drug.

**Results:**

Overall we identified 3,249 reported cases of suicidal/self-injurious behavior or depression, 2,925 (90%) for varenicline, 229 (7%) for bupropion, and 95 (3%) for nicotine replacement. Compared to nicotine replacement, the disproportionality results (OR (95% CI)) were varenicline 8.4 (6.8–10.4), and bupropion 2.9 (2.3–3.7). The disproportionality persisted after excluding reports indicating concomitant therapy with any of 58 drugs with suicidal behavior warnings or precautions in the prescribing information. An additional antibiotic comparison group showed that adverse event reports of suicidal/self-injurious behavior or depression were otherwise rare in a healthy population receiving short-term drug treatment.

**Conclusions:**

Varenicline shows a substantial, statistically significant increased risk of reported depression and suicidal/self-injurious behavior. Bupropion for smoking cessation had smaller increased risks. The findings for varenicline, combined with other problems with its safety profile, render it unsuitable for first-line use in smoking cessation.

## Introduction

Treatments for smoking cessation include counseling without pharmacological intervention (“cold turkey”) and a variety of nicotine replacement products, bupropion (an antidepressant), and varenicline (a partial agonist of α4β2 nicotinic acetylcholine receptors). The U.S. Food and Drug Administration (FDA) has required Boxed Warnings (a.k.a. “Black Box Warnings”) for physicians, and a mandatory Medication Guide for patients regarding serious psychiatric side effects for two pharmacological treatments, varenicline and bupropion [1]. The warnings refer to suicidal behaviors prominently, but also mention depression while noting “Depressed mood may be a symptom of nicotine withdrawal” [2].

Some epidemiological studies report a higher incidence of suicidal thoughts and behaviors among current but not former smokers [3]–[6]. However, much of the elevated risk may be explained by differences in the populations being compared rather than as a possible effect of the nicotine exposure itself. While “dysphoria and depressed mood” is listed among eight possible nicotine withdrawal symptoms in the current Diagnostic and Statistical Manual of Mental Disorders (DSM-IV-TR), [7] little supporting evidence could be found in clinical studies and reviews. These reports provided stronger evidence for other withdrawal symptoms such as irritability, insomnia, and weight gain, [8]–[10] which suggests that depression is not common in nicotine withdrawal.

While suicidal behaviors are not associated with smoking cessation itself, [11], [12] they are linked to at least 58 different approved prescription drugs that have Boxed Warnings, Warnings or Precautions in the package inserts. The list includes varenicline, 23 antidepressant drugs including bupropion, 17 anti-epileptic drugs, and treatments for asthma, viral illness, malaria prevention and acne. Inspection of the warnings for the various compounds reveals that suicidal events were rare in clinical trials. Class warnings for anti-epileptic and antidepressant drugs were based on FDA meta-analyses of numerous clinical trials and were extended to drugs with similar indications or mechanisms of action [13], [14]. Most other warnings were based on adverse event reports.

Tobacco use is responsible for 1 in 5 deaths in the United States each year [15] and adds $193 billion to health care costs. It is among the most treatment-resistant forms of drug dependency, with 36% of the nation's smokers attempting to quit each year but only 3% succeeding for six months or more [16].

In this study we examine the comparative neuropsychiatric safety profiles of varenicline, bupropion and nicotine replacement products with regard to suicidal/self-injurious behavior and depression as reflected in adverse drug event data.

## Methods

### Source Data

Case reports for this study were extracted from a database of all adverse drug event reports received by the FDA since 1998 into its Adverse Event Reporting System (AERS), and released for research use [17]. The case reports are familiar to medical professionals as “MedWatch” reports, the name that appears on the FDA direct reporting form. The reports are either submitted by drug manufacturers, who must forward information about any serious adverse event of which they are informed, or sent directly to the FDA by health professionals or consumers.

As in clinical studies, the event narratives are represented by one or more Preferred Terms drawn from the Medical Dictionary for Regulatory Affairs (MedDRA) [18]. We selected United States case reports coded with a serious health outcome, defined by FDA regulation as death, disability, congenital defect, initial or prolonged hospitalization, a life-threatening event, an event requiring intervention to prevent harm, or other serious outcome [19]. We included Expedited reports from manufacturers but excluded Periodic reports with ambiguous coding of the health outcome “other,” which could mean either “other serious” or “other than serious.” We excluded cases arising from clinical studies and foreign reports, and we also excluded reports explicitly associated with legal claims because of the likelihood that they duplicated cases submitted by others. When one or more follow up reports existed for the same case, the most recent revision was selected. When a report listed multiple health outcomes, the most severe outcome was listed in this priority: death, disability, and other serious events. Report sources were recoded into two categories, health professionals and consumers. The health professional category included physicians, pharmacists, the medical literature, and unspecified “other” health professionals. If both health professionals and consumers were listed as identifiable report sources, the cases were coded as health professionals. Drug names were standardized based on the National Library of Medicine RxNorm [20] ingredient names, and do not distinguish between salts, esters, dosage forms or routes of administration.

### Study Drugs

We selected case reports from 1998 through September 2010 for varenicline (Chantix, Champix), bupropion for smoking cessation (Zyban, or bupropion explicitly indicated for smoking cessation in the adverse event reports). As comparison groups we selected case reports for all nicotine replacement products regardless of route of administration, and the combined case reports for three frequently dispensed antibiotics, amoxicillin, amoxicillin-clavulanate, and azithromycin. Nicotine replacement, typically available without prescription, was selected as a representative comparison group for the patient population trying to stop smoking using drug therapy. To assess the underlying risks that might be attributed to the smoking cessation population independent of study drugs, the antibiotic comparison group was selected to capture reported event rates in a generally healthy population not seeking to stop smoking, in short-term drug treatment, and without a uniformly confounding chronic disease.

### Case Selection

To identify cases of suicidal/self-injurious behavior we utilized the Standardized MedDRA Query (SMQ), narrow scope, for *Suicide/self-injury* to select cases that included any of 11 specified medical terms. To identify depression cases we matched any of the 22 terms in the narrow scope SMQ for *Depression (excluding suicide and self-injury)*. SMQs were developed and tested by the pharmaceutical industry to identify possible cases of various types of adverse reactions. Both MedDRA itself and the SMQs are revised twice a year. This study relies on MedDRA version 13.1 [21]. We also selected the MedDRA Preferred Terms *Headache*, *Pain* or both as an outcome because these are widespread general ailments not associated with any of the study drugs. These terms provided an additional comparison across drugs because we assumed, *a priori*, that these effects should be similar in a similar patient population.

### Concomitant Therapy

Because the case reports frequently indicated that more than one drug was being taken at the time of the reported event, we sought to analyze the influence of concomitant therapy. From product labeling we identified 58 drugs with a Boxed Warning, Warning or Precaution about suicidal behaviors, including varenicline and bupropion (Appendix S1). The texts of the warnings varied widely. The concomitant therapy variable permitted us to identify and exclude case reports indicating the patient had taken other drugs with some form of precaution or warning about suicidal/self-injurious behavior.

### Statistical Analysis

The primary endpoint for this study was every case indicating either suicidal/self-injurious behavior or depression. Because one case report might contain MedDRA terms for both SMQs, a composite endpoint was calculated to eliminate double counting. To compare event rates we created 2×2 tables for each drug and comparator with the endpoint events and all other events for that drug in each column. We used Fisher's exact test to calculate the odds ratio (OR), 95% confidence interval, and test the null hypothesis that the rates did not differ between drugs. To test for the independence of the three smoking cessation drugs and reports of headache/pain we utilized the Yates χ^2^ test.

Results for depression and suicidal/self-injurious behavior were also analyzed separately. The results were also analyzed after excluding cases indicating concomitant use of any of the 58 drugs with warnings or precautions about suicidal or self-injurious behavior.

The master database of all adverse event reports submitted to the FDA was maintained on a MySQL open source database (http://www.mysql.com/) and analyzed with the R Package for Statistical Computing (http://www.r-project.org/).

## Results

### Patient and Event Characteristics

We identified 17,290 case reports meeting the study criteria for all types of serious adverse events, including 13,243 cases in the smoking cessation population and 4,047 in the antibiotic comparison group. In the smoking cessation treatment population, 3,249/13,243 (25%) of the cases reflected either depression, suicidal/self-injurious behavior or both, compared to 48/4,047 (1%) of the cases in the antibiotic group. Table 1 shows the patient and case report characteristics overall for the three smoking cessation drugs and the antibiotic comparison group. While patient and case report characteristics overall were similar among all four groups, a few differences could be observed. As might be expected, antibiotics were used in a wider range of patient age than were the smoking cessation products, as indicated by a greater standard deviation in the mean age. Reporting sources were evenly divided between consumers and health professionals except for nicotine replacement products, for which 83% of case reports came from consumers.

**Table 1:** Characteristics of Adverse Drug Event Reports.

	Varenicline		Bupropion		Nicotine		Antibiotics	
	(N = 9575)		(N = 1751)		(N = 1917)		(N = 4047)	
	N (%)		N (%)		N (%)		N (%)	
Female	5733	(62.5)	874	(51.5)	1123	(60.9)	2064	(51.0)
Age mean (SD),y	49	(12.6)	44	(13.4)	50	(14.3)	50	(25.3)
**Report origin**								
Direct to FDA	2206	(23.0)	199	(11.4)	115	(6.0)	1064	(26.3)
Mfr-Expedited	6717	(70.2)	1313	(75.0)	1757	(91.7)	2813	(69.5)
Mfr-Periodic	652	(6.8)	239	(13.6)	45	(2.3)	170	(4.2)
**Report source**								
Health professional	3153	(40.0)	741	(49.3)	313	(17.1)	1734	(55.3)
Consumer	4726	(60.0)	763	(50.7)	1516	(82.9)	1403	(44.7)
**Health outcome**								
Death, any cause	470	(4.9)	140	(8.0)	111	(5.8)	318	(7.9)
Disability	244	(2.5)	165	(9.4)	41	(2.1)	194	(4.8)
Other serious	8861	(92.5)	1446	(82.6)	1765	(92.1)	3535	(87.3)

The specific medical terms (MedDRA Preferred Terms) that resulted in the classification of a case in either of the two primary SMQs are shown in Table 2. For the suicidal/self-injurious behavior SMQ, suicidal ideation was the most frequent term, appearing in 1,255/2,045 (61%) of cases. However, completed suicide appeared in 298 cases (15%) and suicide attempt in another 388 cases (19%). For the depression SMQ the simple Preferred Term D*epression* appeared in 1,913/2,210 (87%) of selected cases. Among the smoking cessation drugs, the only marked difference in term frequency was that the varenicline cases were coded to a greater variety of different terms than were the bupropion and nicotine products.

**Table 2:** MedDRA terms in suicidal/self-Injurious behavior and depression SMQs.

SUICIDAL/SELF-INJURIOUS BEHAVIOR SMQ								
MedDRA Term	VARENICLINE		BUPROPION		NICOTINE		ANTIBIOTIC	
	n (%)		n (%)		n (%)		n (%)	
Completed suicide	272	(15.0)	19	(12.3)	4	(8.0)	3	(14.3)
Depression suicidal	10	(0.5)	3	(1.9)	0	(0.0)	1	(4.8)
Intentional overdose	31	(1.7)	32	(20.6)	4	(8.0)	8	(38.1)
Intentional self-injury	49	(2.7)	7	(4.5)	1	(2.0)	1	(4.8)
Multiple drug overdose intentional	1	(0.1)	0	(0.0)	0	(0.0)	0	(0.0)
Poisoning deliberate	2	(0.1)	1	(0.6)	0	(0.0)	0	(0.0)
Self-injurious ideation	65	(3.6)	3	(1.9)	0	(0.0)	0	(0.0)
Self injurious behavior	45	(2.5)	0	(0.0)	0	(0.0)	0	(0.0)
Suicidal behavior	63	(3.5)	1	(0.6)	1	(2.0)	0	(0.0)
Suicidal ideation	1135	(62.4)	73	(47.1)	40	(80.0)	7	(33.3)
Suicide attempt	323	(17.8)	56	(36.1)	2	(4.0)	7	(33.3)
Total any term*	1819		155		50		21	

DEPRESSION SMQ								
MedDRA Term	VARENICLINE		BUPROPION		NICOTINE		ANTIBIOTIC	
Adjustment disorder with depressed mood	1	(0.1)	0	(0.0)	0	(0.0)	0	(0.0)
Adjustment disorder with mixed anxiety and depressed mood	1	(0.1)	0	(0.0)	0	(0.0)	0	(0.0)
Anhedonia	38	(1.9)	4	(3.3)	0	(0.0)	0	(0.0)
Decreased interest	33	(1.7)	3	(2.5)	1	(1.7)	0	(0.0)
Depressed mood	180	(9.0)	15	(12.4)	8	(13.8)	2	(6.5)
Depression	1735	(86.8)	102	(84.3)	47	(81.0)	29	(93.5)
Depressive symptom	5	(0.3)	1	(0.8)	0	(0.0)	0	(0.0)
Dysphoria	7	(0.4)	3	(2.5)	0	(0.0)	0	(0.0)
Dysthymic disorder	1	(0.1)	1	(0.8)	1	(1.7)	0	(0.0)
Electroconvulsive therapy	2	(0.1)	0	(0.0)	0	(0.0)	0	(0.0)
Feeling guilty	6	(0.3)	0	(0.0)	0	(0.0)	0	(0.0)
Feeling of despair	43	(2.2)	6	(5.0)	3	(5.2)	0	(0.0)
Feelings of worthlessness	22	(1.1)	1	(0.8)	0	(0.0)	0	(0.0)
Major depression	45	(2.3)	1	(0.8)	2	(3.4)	0	(0.0)
Postpartum depression	1	(0.1)	0	(0.0)	0	(0.0)	0	(0.0)
Total any term*	2000		121		58		31	

*Totals do not add because more than 1 term could appear in a single case report.

### Endpoint Results

**Figure 1:**
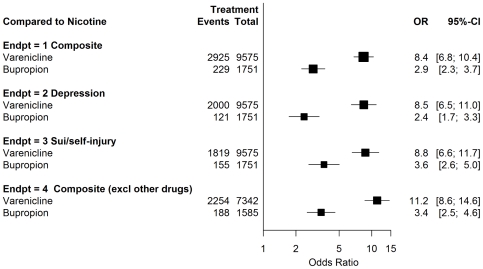
Varenicline, bupriopion versus nicotine replacement.

**Figure 2:**
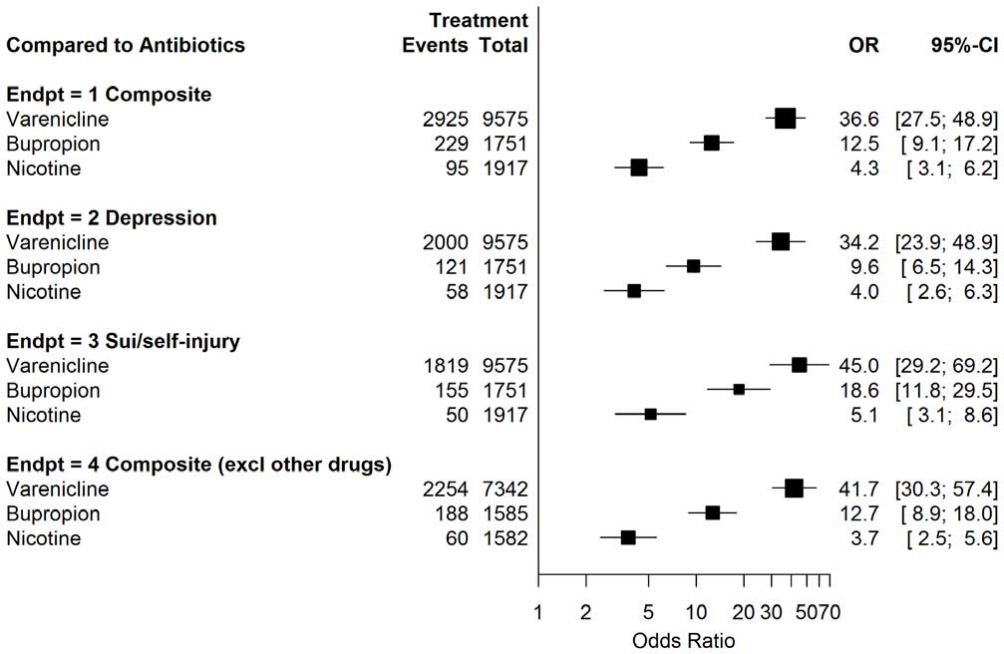
Varenicline, bupropion versus 3 antibiotics.

The odds ratio and 95% confidence intervals for the endpoints in this study are shown in Figures 1 and 2. Figure 1 shows the results compared to nicotine replacement. Varenicline showed disproportional risks, OR 8.4 (CI 6.8–10.4). The odds ratio for bupropion was smaller than for varenicline, but also elevated, OR 2.9 (CI 2.3–3.7). For varenicline compared to bupropion, the odds ratio was 2.9 (CI 2.5–3.4). The results were similar when depression and suicidal/self-injurious behavior were examined separately. Also, 954/3,249 (29%) of the composite endpoint cases fell into both the depression and suicidal/self injurious behavior SMQs. As seen in Figure 2, the data show that compared to the antibiotic comparison group, the OR was elevated for all three smoking cessation treatments, although to different degrees: varenicline OR 36.6 (CI 27.5–48.9), bupropion OR 12.5 (CI 9.1–17.2); nicotine replacement products OR 4.3 (CI 3.1–6.2).

### Concomitant Therapy Drugs

We identified 58 drugs (including bupropion and varenicline) which currently have a Boxed Warning, Warning or Precaution about suicidal behaviors in the prescribing information. Overall, 3,068/17,290 (18%) of all cases included concomitant therapy with another drug with a suicide warning or precaution. The results when these cases were excluded are also shown in Figures 1 and 2. The statistics were similar to the primary endpoint, which did not exclude these cases.

### Headache and Pain

The headache and pain adverse event terms were selected because they are among the most frequent and widely reported, non-specific symptoms both with and without drug therapy, and not clearly associated with any of the study drugs. Headache or pain occurred in 1,032/13,243 (8%) of the smoking cessation cases, but the differences among the three drugs were not statistically significant (χ^2^ = 2.5; df = 2; p = 0.28). However, headache and pain were reported more frequently for the smoking cessation treatment patients than for the antibiotic comparison group OR 1.95 (CI 1.7–2.3). While the difference is small, the result is plausible since nicotine withdrawal could cause these symptoms.

### Unadjusted Event Totals

Even though varenicline was marketed for approximately 4 years of the nearly 13-year study period, it accounted for a disproportionate share of the overall total serious adverse drug events reported in the smoking cessation population, 9,575/13,243 (72%). For the main outcome measure we identified 3,249 reported events of suicidal/self-injurious behavior or depression, 2,925 (90%) for varenicline, 229 (7%) for bupropion, and 95 (3%) for nicotine replacement. For completed suicides, varenicline was associated with 272/295 (92%) of smoking treatment cases, bupropion for 19 cases (6%) and nicotine products for 4 cases (1%). The results were similar for suicide attempts, with 323/381 (85%) of all reported suicide attempts for varenicline, 56 cases (15%) for bupropion, and 2 cases (<1%) for nicotine replacement products.

## Discussion

These data support three conclusions about suicidal/self-injurious behavior and depression in smoking cessation treatment. First, the risks of these reported serious events were higher among nicotine replacement patients compared to a broader population prescribed commonly used antibiotics, suggesting higher risks in the smoking cessation treatment population employing pharmacological assistance. Second, bupropion has some additional excess reported risk when used for smoking cessation compared to the nicotine products. Finally, varenicline has markedly higher reported risk than any of the comparators, a risk that was not reduced regardless of the form of adjustment used. In addition, these findings were consistent with a simpler study using United Kingdom adverse event data [22] and a prospective cohort study in New Zealand [23]. Disproportionality analysis is a proven technique and is increasingly being used to detect associations in adverse event data that escape detection in clinical trials because of their rarity or uncertain event ascertainment [24]–[27]. While the full text narratives of individual case reports were not available for this study, the FDA safety analysis that led to the varenicline and bupropion Boxed Warnings summarized numerous credible individual case narratives of suicidal behavior occurring for the first time in persons with no previous psychiatric history [11].

### Adjusting for Possible Confounding Factors

This analysis seeks to adjust for the following confounding factors: 1) Since adverse event reporting rates might vary over time because of publicity or other factors, we included the entire time interval during which the study drugs had been available since 1998. 2) Because patient exposure varied and reporting rates might theoretically be different, we set the endpoint as the proportion of cases for each specific drug compared to all other serious reports for that same drug. 3) To consider the possibility that the endpoint events might actually be a risk of the underlying patient population we used nicotine replacement as a proxy for the smoking cessation population and compared it to a broader general population exposed to short-term treatment with antibiotics. 4) The evidence that this method identifies true differences between drugs for the primary endpoints is strengthened because the non-specific headache and pain endpoint showed, as expected, no differences among smoking cessation drugs. 5) To reduce possible bias in selection of specific endpoint terms for depression and suicide/self-injury, we utilized without change SMQs created by industry to identify possible cases. 6) We evaluated a possible effect of concomitant therapy by calculating the odds ratios with and without other drugs with existing suicidal behavior warnings and precautions. 7) The antibiotic treatment group, which is not associated with the endpoint, served as an additional check to account for the possibility that any available drug might be taken randomly in overdose in some suicide attempts. While these adjustments substantially improve the precision of the estimates, the character of adverse event data does not permit comparison groups that are similar to the treatment groups in most identifiable characteristics, as can be achieved in some epidemiological studies and randomized controlled trials.

### Clinical and Epidemiological Studies

Increased risk of suicidal/self-injurious behavior was generally not established in clinical trials of varenicline, according to FDA reviews [11], [28] and the sponsor's meta-analysis [29]. However, the meta-analysis reported an increased rate of overall psychiatric side effects when sleep disorders were included, and higher but not statistically significant incidence of depressed mood disorders. Several reasons may explain why suicidal/self-injurious behavior was not apparent in clinical trials prior to approval. The trials were powered to measure efficacy, not less frequent adverse events. Suicidal/self-injurious behaviors are a relatively rare event, with statistically significant excesses of events rarely reported in individual clinical trials for any approved drug. Patient exposure in Phase II/III trials for varenicline was relatively small (n = 3,940), and even smaller at the recommended dose (n = 1,070). The FDA suicidal behavior warnings for antidepressant and anti-epileptic drugs were derived from systematic pooled analyses combining the results of large numbers of clinical trials. Even these large pooled trials had few documented events, and in addition may have underestimated actual risks because few clinical trials used a systematic side effects checklist, relying instead on spontaneously volunteered patient reports at study clinic visits. The FDA concluded in 2008 that the pre-approval varenicline clinical trial data alone were not capable of adequately addressing the issue of suicidal/self-injurious behavior [11]. A medical records database study in the United Kingdom also reported finding no association [30]. However, it was limited by weak event ascertainment, and contradicted by the strong signal observed in that country's adverse event reporting system [22] and by a cohort study in New Zealand [23].

### Limitations of the Study

Submission of a case report does not in itself prove that the suspect drug caused the event observed. In addition, it was not possible to evaluate each individual case report according to any of the commonly employed tools to assess issues such as time of onset, possible alternative causes, effects of discontinuation, rechallenge, and report quality. On the other hand, the total number of cases was large, sustained over substantial periods of time, and found in both direct-to-FDA and manufacturer reports. While these data provide statistically significant evidence of an association between varenicline and the study endpoints, it would take a different study design with an appropriate denominator of users to estimate the incidence of these adverse effects. In this study we used nicotine replacement patients as representative of the population seeking to quit smoking with pharmacological assistance. However, we cannot exclude the possibility that nicotine replacement might slightly increase or decrease the risk of reported suicidal/self-injurious behavior or depression. Previous nicotine studies [31] and the relatively small number of reported events compared to the nearly universal availability of these OTC nicotine products suggest that the drug effects, if any, are small. The number of adverse event cases for bupropion was likely undercounted because information about the indication for treatment was missing for many adverse event reports and this study selected only cases with specific information that bupropion was prescribed for smoking cessation. The risks for varenicline were understated in these data because the drug was only marketed for approximately 4 years of the nearly 13-year study period, and because of the possibility that manufacturer coding problems may have led to an undercount [32]. In addition, while reporting rates for these side effects are not known, only a small fraction of adverse events that occur are reported in a voluntary system.

### Treatment Efficacy

The efficacy of the three major pharmacological interventions for smoking cessation has been studied in numerous randomized clinical trials [33], [34]. One analysis focusing on longer-term outcomes reported odds ratios compared to placebo ranging from 1.7 for nicotine gum to 2.4 for varenicline [33]. Retrospective studies of successful quitters often favor self quitters or cold turkey approaches [35]. The FDA evaluation of the varenicline trials showed that the drug had superior quit rates at 12 weeks; but by 52 weeks a large majority in all groups had resumed smoking—only approximately 25–27% of varenicline patients had remained largely abstinent compared to 17–19% of bupropion patients and 9–12% of the placebo group [36]. The only published trial comparing varenicline to nicotine patches found no statistically significant difference in reported 7-day abstinence at 52 weeks [37].

### Other Safety Concerns

While suicidal/self-injurious behavior and depression appear to be prominent side effects of varenicline, they are by no means the only safety issues. Varenicline has been associated with aggression and violence in three studies [25], [27], [38] and carries a warning about this behavior. Its effect on vision, cognition, and motor control and other risks have led to its being banned for airline pilots, air controllers, military pilots and missile crews, and restricted for truck drivers [39]. Varenicline is also associated with an increase in the risk of serious cardiovascular events [40]. In addition, it is associated with hypersensitivity, angioedema and potentially life-threatening severe cutaneous adverse events [2].

### Unanswered Questions

While a growing body of research from multiple sources establishes that varenicline substantially increases the risk of psychiatric side effects, it remains uncertain how frequently these events occur. Estimating incidence rates would require a large cohort, a validated psychiatric symptoms checklist, and sufficient power over one year to detect event rates that may be as low as 1 or 2 per 1,000 but could be more than 1 in 100. The same study could also assess cardiovascular, accident, and other known risks. Even better incidence estimates will not address the value judgment of how to weigh the possible benefits of 52 weeks of smoking abstinence for 1 or 2 out of every 10 patients treated against the risk of less frequent adverse events such as violent and suicidal behavior that can have immediate, catastrophic and irreversible effects on self, family, and career. In the meantime, safer alternatives now exist and should be preferred.

### Clinical and Regulatory Implications

The overall safety profile of varenicline makes it unsuitable for first-line use in smoking cessation. We agree with the recommendation of the U.S. Veterans Administration (VA) [41] that varenicline should be prescribed only after failure of nicotine replacement, bupropion, or a combination. The VA also recommends a mental status examination to assess risk of suicidal or violent behavior prior to prescribing varenicline. In addition, varenicline should not be prescribed for sensitive occupations such as airline pilots, air controllers, active duty military, police officers, truck and bus drivers, and emergency medical workers. Also, the FDA should consider revising the suicidal behavior and depression language in the Boxed Warning and Highlights of Prescribing Information to state clearly that the risks of suicidal behavior and depression are higher with varenicline than with other smoking cessation treatments.

In conclusion, varenicline shows a substantial, statistically significant increased risk of reported depression and suicidal/self-injurious behavior. The excess risk persisted even after adjusting for numbers of patients exposed, for the possibility of different reporting rates, and for concomitant therapy. Bupropion for smoking cessation had increased risks but less so than for varenicline. The findings for varenicline, combined with other problems with its safety profile, render it unsuitable for first-line use in smoking cessation.

## Supporting Information

Appendix S1 Drugs with Suicidal Behavior Warnings.
(DOC)

## References

[1] United States Food and Drug Administration (2009). Chantix and Zyban to Get Boxed Warning on Serious Mental Health Events.

[2] Pfizer Inc. (2010). CHANTIX (varenicline tartrate) tablet, film coated [package insert].

[3] Miller M, Hemenway D, Bell NS, Yore MM, Amoroso PJ (2000). Cigarette smoking and suicide: a prospective study of 300,000 male active-duty Army soldiers.. Am J Epidemiol.

[4] Leistikow BN, Martin DC, Samuels SJ (2000). Injury death excesses in smokers: a 1990–95 United States national cohort study.. Inj Prev.

[5] Kessler RC, Berglund PA, Borges G, Castilla-Puentes RC, Glantz MD (2007). Smoking and suicidal behaviors in the National Comorbidity Survey: Replication.. J Nerv Ment Dis.

[6] Breslau N, Schultz LR, Johnson EO, Peterson EL, Davis GC (2005). Smoking and the risk of suicidal behavior: a prospective study of a community sample.. Arch Gen Psychiatry.

[7] American Psychiatric Association and Task Force on DSM-IV (2000). Diagnostic and Statistical Manual of Mental Disorders DSM-IV-TR.

[8] Hatsukami DK, Dahlgren L, Zimmerman R, Hughes JR (1988). Symptoms of tobacco withdrawal from total cigarette cessation versus partial cigarette reduction.. Psychopharmacology (Berl).

[9] Hughes JR (2007). Effects of abstinence from tobacco: valid symptoms and time course.. Nicotine Tob Res.

[10] Hughes JR (2007). Depression during tobacco abstinence.. Nicotine Tob Res.

[11] Pollock M, Lee J, Mosholder AD (2008). Suicidality: Varenicline, bupropion, nicotine transdermal patch.

[12] Hughes JR (2008). Smoking and suicide: a brief overview.. Drug Alcohol Depend.

[13] Mosholder AD, Willy M (2006). Suicidal adverse events in pediatric randomized, controlled clinical trials of antidepressant drugs are associated with active drug treatment: a meta-analysis.. J Child Adolesc Psychopharmacol.

[14] Levenson M, Rochester G (May 2008). Statistical Review and Evaluation: Anti-epileptic Drugs and Suicidality.

[15] National Center for Chronic Disease Prevention and Health Promotion, Office on Smoking and Health (2010). How Tobacco Smoke Causes Disease: The Biology and Behavioral Basis for Smoking-Attributable Disease: A Report of the Surgeon General.

[16] National Cancer Institute, Centers for Disease Control and Prevention (2009). National Cancer Institute and Centers for Disease Control and Prevention Co-sponsored Tobacco Use Supplement to the Current Population Survey (2006–07).

[17] United States Food and Drug Administration (2010). The Adverse Event Reporting System (AERS): Latest Quarterly Data File.

[18] MedDRA Maintenance and Support Services Organization (2010). Introductory Guide MedDRA Version 13.1.

[19] United States Food and Drug Administration (2010). Code of Federal Regulations Title 21, Section.314.80 Postmarketing reporting of adverse drug experiences.

[20] National Library of Medicine (2010). Unified Medical Language System (UMLS): RxNorm.

[21] MedDRA Maintenance and Support Services Organization (2010). Introductory Guide for Standardized MedDRA Queries (SMQs) Version 13.1.

[22] Moore TJ, Furberg CD (2009). Varenicline and suicide. Risk of psychiatric side effects with varenicline [letter].. BMJ.

[23] Harrison-Woolrych M, Ashton J (2011). Psychiatric adverse events associated with varenicline: an intensive postmarketing prospective cohort study in New Zealand.. Drug Saf.

[24] Elashoff M, Matveyenko AV, Gier B, Elashoff R, Butler PC (2011). Pancreatitis, pancreatic, and thyroid cancer with glucagon-like peptide-1-based therapies.. Gastroenterology.

[25] Rouve N, Bagheri H, Telmon N, Pathak A, Franchitto N (2011). Prescribed drugs and violence: a case/noncase study in the French PharmacoVigilance Database.. Eur J Clin Pharmacol.

[26] Wallerstedt SM, Brunlof G, Sundstrom A, Eriksson AL (2009). Montelukast and psychiatric disorders in children.. Pharmacoepidemiol Drug Saf.

[27] Moore TJ, Glenmullen J, Furberg CD (2010). Prescription drugs associated with reports of violence towards others.. PLoS One.

[28] Josefberg H (2006). Clinical Safety Review Application 21-928 Varenicline tartrate.

[29] Tonstad S, Davies S, Flammer M, Russ C, Hughes J (2010). Psychiatric adverse events in randomized, double-blind, placebo-controlled clinical trials of varenicline: a pooled analysis.. Drug Saf.

[30] Gunnell D, Irvine D, Wise L, Davies C, Martin RM (2009). Varenicline and suicidal behaviour: a cohort study based on data from the General Practice Research Database.. BMJ.

[31] Greenland S, Satterfield MH, Lanes SF (1998). A meta-analysis to assess the incidence of adverse effects associated with the transdermal nicotine patch.. Drug Saf.

[32] United States Food and Drug Administration (2011). Chantix: QuarterWatch Article.

[33] Eisenberg MJ, Filion KB, Yavin D, Belisle P, Mottillo S (2008). Pharmacotherapies for smoking cessation: a meta-analysis of randomized controlled trials.. CMAJ.

[34] Silagy C, Lancaster T, Stead L, Mant D, Fowler G (2004). Nicotine replacement therapy for smoking cessation.. Cochrane Database Syst Rev.

[35] Chapman S, MacKenzie R (2010). The global research neglect of unassisted smoking cessation: causes and consequences.. PLoS Med.

[36] Winchell C (2006). Supervisory Review of NDA: Safety Review and Primary Clinical Efficacy Review Varenicline Pfizer Inc.U.S. Food and Drug Administration, Center for Drug Evaluation and Research.

[37] Aubin HJ, Bobak A, Britton JR, Oncken C, Billing CB (2008). Varenicline versus transdermal nicotine patch for smoking cessation: results from a randomised open-label trial.. Thorax.

[38] Moore TJ, Glenmullen J, Furberg CD (2010). Thoughts and acts of aggression/violence toward others reported in association with varenicline.. Ann Pharmacother.

[39] Moore TJ, Cohen MR, Furberg CD (2011). Signals for Varenicline, Levofloxacin and Fentanyl.

[40] Singh S, Loke YK, Spangler JG, Furberg CD (2011). Risk of serious adverse cardiovascular events associated with varenicline: a systematic review and meta-analysis.. CMAJ.

[41] United States Department of Veterans Affairs Pharmacy Benefits Management Services (2011). Clinical Guidance: Varenicline Criteria for Prescribing.

