# Supplementary material for: Suicidal Behavior and Depression in Smoking Cessation Treatments
Source: PLoS One. 2011 Nov 2;6(11):e27016. doi: 10.1371/journal.pone.0027016 (PMC3206890; doi:10.1371/journal.pone.0027016)
Supplement: Appendix S1 — Drugs with Suicidal Behavior Warnings. (DOC) [file pone.0027016.s001.doc]

**Appendix S1 Marketed drugs with suicidal behavior warnings or precautions.**

**Smoking Cessation**

bupropion (Zyban)

varenciline (Chantix)

**Antidepressants**

Selective serotonin reuptake inhibitors (SSRIs)

citalopram (Celexa)

escitalopram (Lexapro)

paroxetine (Paxil)

fluoxetine (Prozac)

fluvoxamine (Luvox)

sertraline (Zoloft)

Serotonin-norepinephrine reuptake inhibitors (SNRIs)

desvenlafaxine (Pristiq)

duloxetine (Cymbalta)

venlafaxine (Effexor)

Norepinephrine-dopamine reuptake inhibitors (NDRIs)

bupropion (Wellbutrin)

Tricyclic antidepressants (TCAs)

amitriptyline (Elavil)

clomipramine (Anafranil)

desipramine (Norpramin)

doxepin (Adapin, Sinequan)

imipramine (Tofranil)

nortriptyline (Pamelor)

protriptyline (Vivactil)

trimipramine (Surmontil)

Hetrocyclic antidepressants

mirtazapine (Remeron)

trazadone (Desyrel, Oleptro)

Monoamine oxidase inhibitors (MAOIs)

isocarboxazid (Marplan)

phenelzine (Nardil)

tranylcypromine (Parnate)

**Benzodiazepine Anti-Anxiety Agents/Sedative-Hypnotics**

alprazolam (Xanax)

clonazepam (Klonopin)

clorazepate (Tranxene)

temazepam (Restoril)

triazolam (Halcion)

**Sedative-Hypnotics**

eszopiclone (Lunesta)

zaleplon (Sonata)

zolpidem (Ambien)

**Antipsychotics**

aripaprazole (Abilify)

quetiapine (Seroquel)

fluoxetine; olanzapine (Symbyax)

**ADHD Medication**

atomoxetine (Strattera)

**Anti-Epileptic Drugs/Mood Stabilizers**

carbamazepine (Tegretol)

divalproex sodium (Depakote)

ethosuximide (Zarontin)

felbamate (Felbatol)

gabapentin (Neurontin)

lamotrigine (Lamitcal)

levetiracetam (Keppra)

methsuximide (Celontin)

oxcarbazepine (Trileptal)

phentoin (Dilantin)

pregabalin (Lyrica)

primidone (Mysoline)

tiagabine (Gabatril)

topiramate (Topamax)

trimethadione (Tridione)

valproic acid (Depekene, Stavzor)

zonisamide (Zonegran)

**Anti-Parkinson’s Agents**

amatadine (Symadine, Symmetrel)

**Interferons**

interferon alfa (Roferon-A, Intron-A, Pegasys, Infergen)

**Acne Medication**

isotretoin (Accutane, Amnesteem, Claravis, Sotret)

**Leukotriene Antagonist**

montelukast (Singulair)

**Malaria Preventive**

mefloquine (Lariam)
